# Injectable hyaluronic acid hydrogels encapsulating drug nanocrystals for long‐term treatment of inflammatory arthritis

**DOI:** 10.1002/btm2.10245

**Published:** 2021-09-15

**Authors:** Yongsheng Gao, Douglas Vogus, Zongmin Zhao, Wei He, Vinu Krishnan, Jayoung Kim, Yujie Shi, Apoorva Sarode, Anvay Ukidve, Samir Mitragotri

**Affiliations:** ^1^ School of Engineering and Applied Sciences Harvard University Cambridge Massachusetts USA; ^2^ Wyss Institute of Biologically Inspired Engineering Boston Massachusetts USA

**Keywords:** arthritis, camptothecin, drug nanocrystals, hyaluronic acid, hydrogels

## Abstract

Antiproliferative chemotherapeutic agents offer a potential effective treatment for inflammatory arthritis. However, their clinical application is limited by high systemic toxicity, low joint bioavailability as well as formulation challenges. Here, we report an intra‐articular drug delivery system combining hyaluronic acid hydrogels and drug nanocrystals to achieve localized and sustained delivery of an antiproliferative chemotherapeutic agent camptothecin for long‐term treatment of inflammatory arthritis. We synthesized a biocompatible, in situ‐forming injectable hyaluronic acid hydrogel using a naturally occurring click chemistry: cyanobenzothiazole/cysteine reaction, which is the last step reaction in synthesizing *D*‐luciferin in fireflies. This hydrogel was used to encapsulate camptothecin nanocrystals (size of 160–560 nm) which released free camptothecin in a sustained manner for 4 weeks. In vivo studies confirmed that the hydrogel remained in the joint over 4 weeks. By using the collagen‐induced arthritis rat model, we demonstrate that the hydrogel‐camptothecin formulation could alleviate arthritis severity as indicated by the joint size and interleukin‐1β level in the harvested joints, as well as from histological and microcomputed tomography evaluation of joints. The hydrogel‐nanocrystal formulation strategy described here offers a potential solution for intra‐articular therapy for inflammatory arthritis.

## 1 INTRODUCTION

Rheumatoid arthritis (RA), a type of inflammatory arthritis, is a chronic autoimmune disease that affects ~1% of the population in developed countries with an average annual medical expenditure of $11,120 per patient.^1^, ^2^ Although great progress has been made over the last two decades, there is still no cure for RA. Also, considerable concerns exist regarding the efficacy and safety of the currently used medications.^3^, ^4^ Even for the lead disease‐modifying anti‐rheumatic drug, methotrexate, only 20%–40% of RA patients have a good clinical response,^4^ and its long‐term use is limited by systemic toxicity.^5^ Therefore, the development of new treatments against RA as well as other inflammatory arthritis represents a major unmet need.

The tumor‐like aggressive proliferation of synoviocytes plays a central role in the propagation of RA,^6^, ^7^ responsible for initiating the inflammatory process and synovial invasion, promoting angiogenesis, and creating pannus tissue.^6^, ^8^ Inhibiting the proliferation of synoviocytes is thus a potential treatment strategy for RA.^9^ Various antiproliferative drugs, developed for cancer treatment, have been shown to be effective to treat symptoms of RA, leading to a convoluted connection between cancer chemotherapeutics and RA treatment.^10^ Camptothecin (CPT), a topoisomerase I inhibitor,^11^ for instance, is one such chemotherapeutic agent that can inhibit the proliferative processes in RA (e.g. synovitis and angiogenesis).^12^, ^13^ However, the in vivo application of this potent drug is limited by its extremely low solubility and instability in biological fluids and the associated high systemic toxicity. Additional obstacles for the application of CPT in inflammatory arthritis treatment, as for other small molecular drugs, are low joint bioavailability and short joint residence time.^14^, ^15^

Localized drug administration, namely intra‐articular (IA) injection, has been demonstrated to be effective in improving drug's joint bioavailability and reducing systemic toxicity.^14^, ^16^ However, extra efforts have to be made to prolong the drug's residence time in the joint, given that small molecular drugs are typically cleared into systemic circulation within a few hours or less.^14^ One approach toward that end is to formulate therapeutic agents into macroscopic carriers, such as polymeric microparticles, which are injectable but sufficiently large to avoid the rapid lymphatic drainage.^15^ Zilretta™ (FX006), recently approved by the U.S. Food and Drug Administration (FDA), is one example that uses poly(lactic‐co‐glycolic acid) microspheres to encapsulate triamcinolone acetonide for IA injection.^17^ While polymer particles can efficiently extend the drug release over weeks to months, the drug loading capacity is generally low (usually less than 10 wt%),^18^ and as a result, a large amount of particles has to be injected into the joint to achieve therapeutic drug concentrations.^19^, ^20^, ^21^

An alternative method to creating drug depot inside the joint is to use injectable hydrogels. Hydrogels with three‐dimensional cross‐linked network, high water content, and tissue‐like properties offer a versatile drug delivery platform with tunable drug loading capacity and release kinetics. Hyaluronic acid (HA) hydrogels, in particular, have a track record of safe and effective clinical uses in IA therapy with various injectable HA hydrogels been approved by the FDA to provide viscosupplementation.^14^ Yet, engineering hydrogels that can sustain the release of small molecular drugs in the time frames needed for treating chronic diseases such as inflammatory arthritis is still a difficult task. Encapsulating solid‐form drugs, such as drug nanocrystals, instead of free drugs, can significantly extend the release period. Drug nanocrystals, as commonly used to formulate poorly water‐soluble therapeutical agents, have slow dissolution rates and improved drug stability under physiological conditions.^22^, ^23^ Moreover, the compact crystalline structures are composed of nearly 100% active pharmaceutical ingredient, thus leading to a high loading efficiency. The vast potential of drug crystals in drug delivery has been gradually uncovered, with a few tens of crystalline drug formulations successfully translated to the clinic.^22^ However, their applications in IA drug delivery remain largely unexplored, with only a few drug nanocrystal‐based formulations reported for osteoarthritis treatment.^24^, ^25^

**FIGURE 1:**
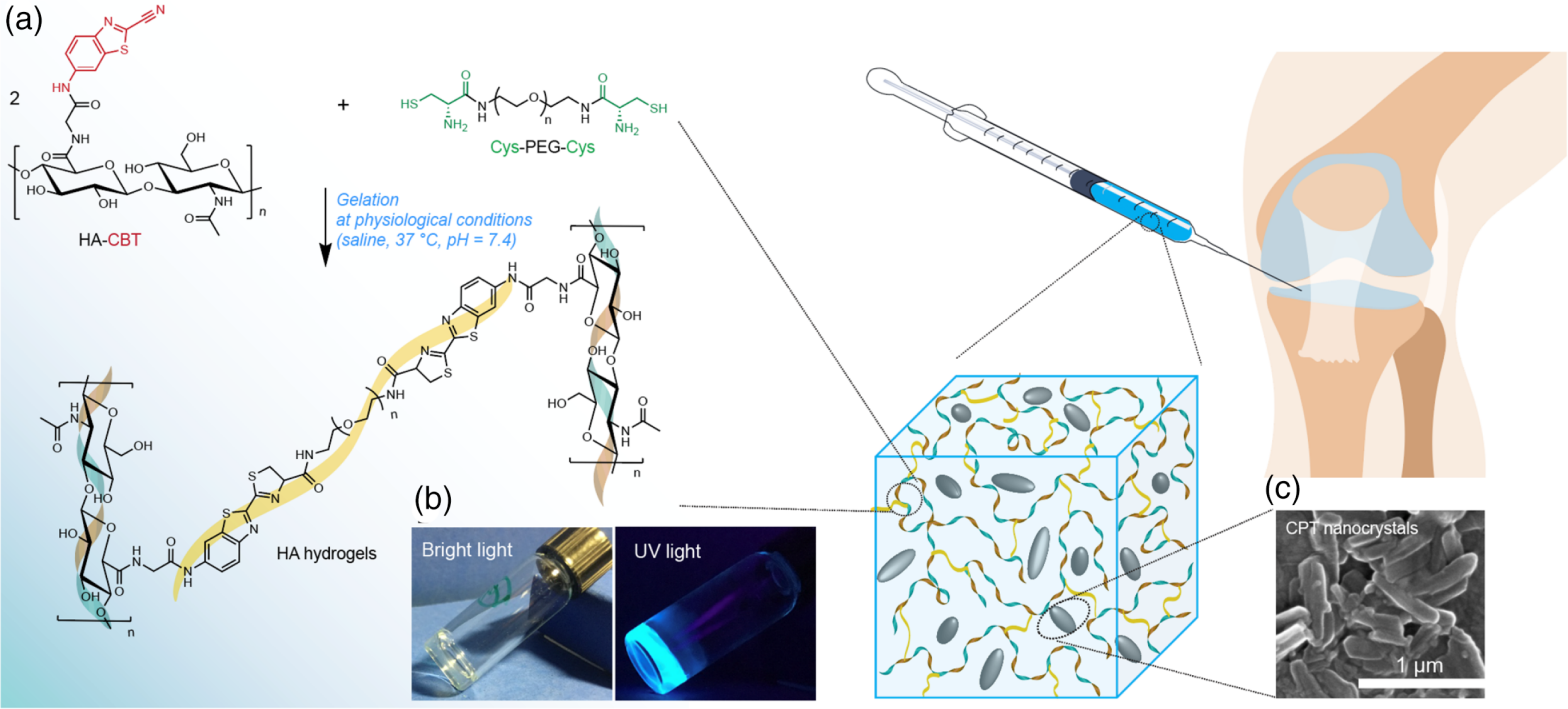
Drug nanocrystal‐loaded HA hydrogels for intra‐articular injection. (a) Gelation reaction scheme between CBT‐conjugated HA (HA‐CBT) and cysteine functionalized PEG linker (Cys‐PEG‐Cys) under mild physiological conditions (neutral pH, aqueous solution and 37°C) without catalyst needed and byproduct generated. (b) The resulting hydrogels are transparent under bright light, and show fluorescence under UV light (360 nm) due to the existence of luciferin moieties. (c) Drug‐nanocrystals can be encapsulated into the hydrogel during the gelation process, and slowly dissolve after intra‐articular injection

Here, we report a nanocrystal‐encapsulated, HA hydrogel‐based formulation for localized, long‐term IA drug delivery (Figure 1), as such to develop a potential inflammatory arthritis treatment by using the chemotherapeutic agent CPT. To the best of our knowledge, this is the first attempt to explore IA injection of nanocrystal‐loaded hydrogels as a sustained release depot for inflammatory arthritis treatment. The first and foremost step toward this is to develop an in situ‐forming injectable HA hydrogel that can load drug nanocrystals. While several HA products are used for IA delivery,^26^, ^27^ the prevalent cross‐linking methods using butanediol‐diglycidyl ether or divinyl sulfone require hostile reaction conditions (e.g. basic pH) and post‐gelation washing processes,^27^, ^28^ which limit their use for in situ gelling and drug encapsulation. Here, we use a naturally occurring, highly biocompatible click reaction: cyanobenzothiazole (CBT)/cysteine reaction as the cross‐linking chemistry, which is ideally suited for this purpose as it has fast kinetics and good yield under physiological conditions without the necessity of catalyst or harsh conditions.^29^, ^30^, ^31^ CPT nanocrystals can be encapsulated into HA hydrogels by simply mixing them with the hydrogel precursors. The promise of CPT nanocrystal/HA hydrogel formulation in achieving long‐term localized IA drug delivery was verified both in vitro and in vivo.

## 2 RESULTS

### 2.1 Chemical synthesis and characterization of HA hydrogel precursors

**FIGURE 2:**
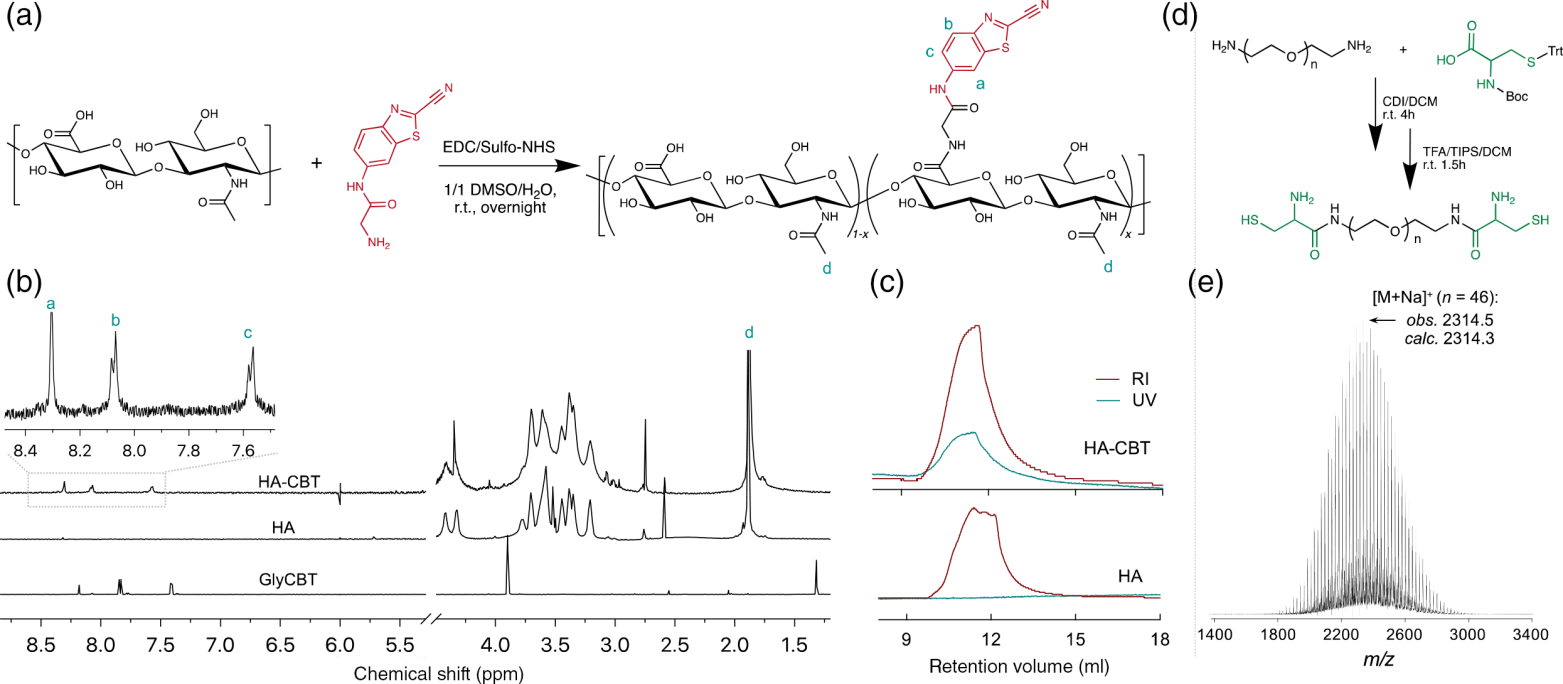
Synthesis and molecular characterization of HA‐CBT (a,b,c) and Cys‐PEG_*n*_‐Cys (d,e). (a) The conjugation of Gly‐CBT to HA was achieved using EDC/Sulfo‐NHS chemistry in 1:1 DMSO:water at room temperature overnight. (b) ^1^H NMR (D_2_O, 600 MHz) spectrum verifies the existence of CBT moieties in the final products. (c) GPC profiles further show the appearance of UV (330 nm) absorbance at the elution time of HA‐CBT (RI detector), in contrast to the blank HA samples, and the similar retention volume of HA‐CBT and HA further demonstrates that no observable degradation of HA molecules occurred during the conjugation and purification process. (d) Reaction scheme for Cys‐PEG_*n*_‐Cys synthesis. (e) MALDI‐TOF‐MS corroborates the proposed molecular structure

An ideal cross‐linking method for fabricating in situ‐forming injectable HA hydrogels that can encapsulate drug nanocrystals should operate under mild physiological conditions (neutral pH in aqueous solution at 37°C) with desirable gelation kinetics and outstanding biocompatibility, such as catalyst‐free, no undesired byproducts, and low toxicity of the initial, final, and degraded products. The cross‐linking chemistry chosen here is the CBT/cysteine reaction, which is the last step reaction in the generation of D‐luciferin in fireflies.^29^, ^30^, ^31^ For the ease of synthesis, we conjugated CBT to HA, which improved its aqueous solubility while preserving cross‐linkability when mixed with cysteine‐based cross‐linkers in aqueous media at a physiological pH. The primary amine of glycine(Gly)‐CBT was coupled to HA carboxyl groups using EDC/sulfo‐NHS chemistry (Figure 2a). Successful conjugation was verified with ^1^H nuclear magnetic resonance (NMR) spectroscopy as the three characteristic CBT proton peaks at *δ* 7.5–8.4 ppm are shown in the final product (Figure 2b). To further confirm the covalent attachment of the Gly‐CBT to HA and the structural integrity of HA after conjugation, aqueous gel permeation chromatography (GPC) with refractive index (RI) and ultraviolet (UV) detectors was used to characterize the HA‐CBT conjugates (Figure 2c). HA‐CBT conjugates have a similar retention volume, that is, similar hydrodynamic volume, to blank HA (the starting material), indicating that the conjugation and purification processes do not degrade HA. This is highly desirable for its further biomedical applications as the biological functions of HA are substantially sensitive to its molecular size.^32^ Moreover, in contrast to blank HA with no UV absorbance, the eluted HA‐CBT products have an observable UV absorbance peak in the similar wavelength range of CBT derivatives (Figure S1), which is a strong evidence that the CBT moieties are covalently attached to the HA backbone. The degree of CBT conjugation (DC) was estimated by comparing the peak areas of aromatic protons from CBT (*δ* 7.5–8.4 ppm) and methyl groups in the acetamido moiety of HA (*δ* 1.7–2.0 ppm), and DC values of 5.1 ± 0.5%, 10.3 ± 0.5%, and 18.5 ± 4.4% were achieved by controlling the molar feed ratio of CBT to carboxyl groups of HA, which is highly beneficial for tuning the hydrogel mesh sizes and mechanical properties. Cysteine functionalized PEG molecules (Cys‐PEG_*n*_‐Cys) were designed as hydrophilic cross‐linkers, which were synthesized by reacting PEG diamines (of different molecular weights) with thiol‐ and amine‐protected cysteine (Figure 2d). PEG was chosen due to its biocompatibility, aqueous solubility, low viscosity, and commercial availability in different sizes.^33^ The structures of the deprotected and purified PEG linkers were confirmed by matrix‐assisted laser desorption ionization time‐of‐flight mass spectrometry (MALDI–TOF‐MS) and ^1^H NMR (Figure 2e; Figures S2 and S3).

### 2.2 Rheological evaluation and optimization of HA hydrogels

**FIGURE 3:**
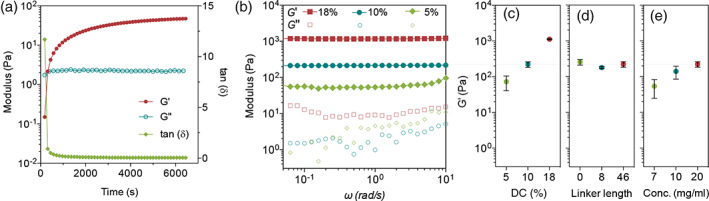
HA hydrogel characterization. Gelation time (a), modulus (b), and the change of storage modulus with (c) HA‐CBT conjugation degree, (d) different Cys‐PEG_*n*_‐Cys linkers (averaged *n* of 0, 8, and 46), and (e) polymer concentration

The kinetics of gelation and final gel properties were then studied and optimized. To correlate their performance under physiological conditions, all reactions and tests were performed at 37°C, unless stated otherwise. Rapid gelation was observed after mixing HA‐CBT conjugates (20 mg/ml) with an equimolar number of Cys‐PEG_*n*_‐Cys linkers in saline. The gelation time was first evaluated through a standard time sweep test using an oscillatory rheometer, where the storage and loss moduli (*G*′ and *G*″, respectively) were recorded as time advances. The cross‐over of *G*′ with *G*″ as an indicator of the gel point occurred at around 300 s (Figure 3a). This falls into the ideal optimal gelation time range (1–10 min)^34^ for an injectable hydrogel. A continuous increase in storage modulus with time after the gel point was reached and indicates the occurrence of post‐gelation cross‐linking reactions. This suggests that the initially formed network percolating the entire system can be further strengthened through such continued cross‐linking. This is indeed beneficial for a practical implementation of hydrogel injections, as the initial formed, loosely cross‐linked hydrogels (low *G*′) can restrict the undesired massive spread of injected polymers, but still permit its injectability (even if the gelation happened inside the syringe) and fillability for tissue cavity with arbitrary shapes. The formation of a well‐structured hydrogel network with solid‐like properties can also be corroborated from the dramatic decrease of tan(*δ*) upon gelation and the large *G*′/*G*″ ratio after gel formation (Figure 3a). Furthermore, as shown in Figure 3b, a constant *G*′ was obtained over the entire frequency range tested, and it is higher than *G*″ by 1–2 orders of magnitude. The independence of *G*′ on the frequency is another characteristic viscoelastic response of a cross‐linked gel network.

According to the polymer network theory, *G*′ is highly dependent on the density of cross‐linking points and chain entanglements, that is, mesh size, which also largely determines the drug release behavior by regulating the diffusion of molecules or particulate carriers.^35^ Thus, we next investigated the tunability of *G*′ of the newly synthesized HA hydrogels. Changing the DC of HA‐CBT and length of Cys‐PEG_*n*_‐Cys linkers are two straightforward ways to control the mesh size. HA‐CBT with DC of 5.1 ± 0.5%, 10.3 ± 0.5%, and 18.5 ± 4.4% were synthesized and cross‐linked by Cys‐PEG_46_‐Cys at 20 mg/ml in saline. As expected, the resulting hydrogels show increased *G*′ with an increase of DC, and a maximum *G*′ value of around 1 kPa was obtained at DC of ~18.5% (Figure 3c). However, since the high degree of modification decreases the hydrophilicity of the overall HA‐CBT conjugates and could raise biosafety concerns, we kept the DC of HA‐CBT conjugates at ~10%. Unlike the DC effect, *G*′ is not sensitive to the linker length (the PEG repeat number *n* of 0, 8, and 46; Figure 3d), which could be attributed to the existence of physical entanglements between HA chains under in situ gelling conditions. Chain entanglements, serving as another type of network junctions other than chemical cross‐links, dominate the overall *G*′.^36^, ^37^ The change of polymer concentration is another way to tune the overall cross‐linking density and a modest increase in *G*′ was observed with an increase in polymer concentration from 7 to 20 mg/ml (Figure 3e). Based on the gelation time and the overall gel properties, we set ~10% DC of HA‐CBT, Cys‐PEG_46_‐Cys and 20 mg/ml concentration as our gelation condition for the remaining studies.

### 2.3 Evaluation of HA hydrogels for sustained drug delivery

**FIGURE 4:**
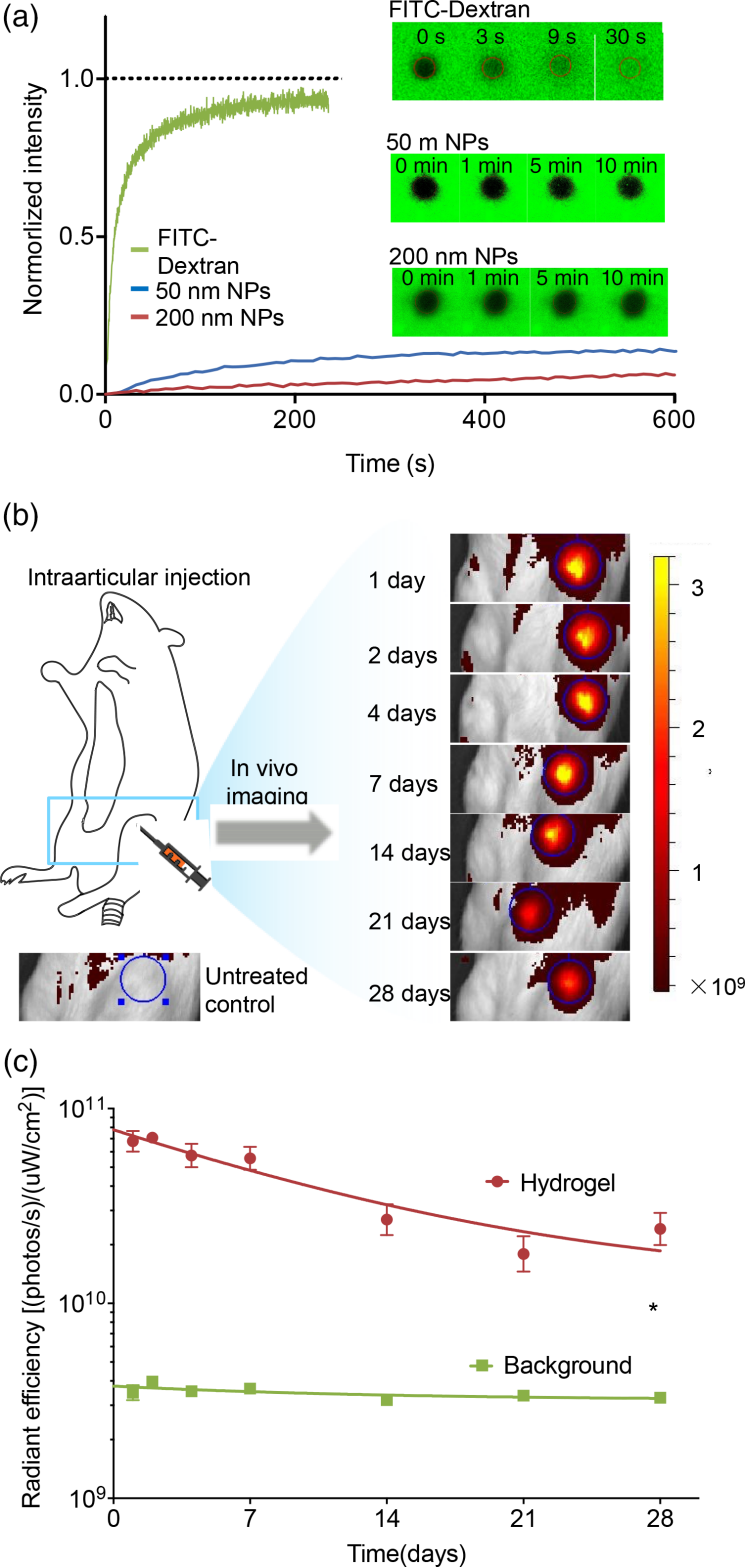
The evaluation of hydrogels for drug delivery. (a) The diffusion properties of the polystyrene nanoparticles inside the hydrogels were characterized using FRAP and compared with FITC‐dextran. (b) Schematic illustration of intra‐articular injection and representative IVIS images of the injected knee joints of rats over 28 days. HA hydrogel was labeled with Alexa Fluor 647. The blue circles with a fixed diameter outlined the ROI range for the quantification of fluorescence. Color scale: 1.6 × 10^8^–3.2 × 10^9^ Radiant efficiency unit: (p/s•cm^2^•sr)/(μW/cm^2^). Noninjected rats were imaged at all the time points to collect the background fluorescent intensity (blue line in c). (c) The joint residence time of fluorescent labeled HA hydrogels. Data points were fit by a one‐phase exponential decay models. Each value represents the mean ± *SEM* (*n* = 5) and statistical analysis by the nonparametric Wilcoxon matched‐pairs signed ranks test (*p < 0.05)

To evaluate their potential in drug delivery, we first measured the mesh size of the optimized HA hydrogels based on the blob model, swelling ratio, and storage modulus (Equations S1–S3, Figure S4).^36^ The estimated mesh size value was ~36 nm. Such mesh size is small enough to efficiently encapsulate nanosized particles or crystals. To directly assess this, the diffusion of nanoparticles inside the hydrogels was evaluated by fluorescence recovery after photobleaching (FRAP) method (Figure 4a). Free FITC‐dextran molecules (Mw 70 kDa) used as control here, shows full recovery soon after photobleaching with a diffusion coefficient of 7.35 ± 0.46 μm^2^/s. Commercial fluorescent polystyrene nanoparticles with a size of 200 nm were encapsulated in hydrogels and subjected to the FRAP test. After 10 min, only 6.5% fluorescent intensity was recovered, demonstrating that nanoparticles are firmly immobilized inside the gel network with limited diffusivity. Similarly, 50 nm nanoparticles showed a similarly slow recovery behavior, with only 13.9% recovery after 10 min. A similar result was reported previously by Seiffert et al. which is attributed to the special translational mobilities of particles in polymer matries.^38^ The ability to immobilize nanosized particles within the hydrogel network is indeed highly desirable for constructing drug nanocrystal depots.

Next, we evaluated the stability of hydrogel materials inside the synovial fluid. Rapid clearance and thus short joint residence time of the delivered substance represent the most challenging obstacle for IA therapy.^14^, ^15^ The stability of the IA‐injected HA hydrogels is thus crucial for the ultimate performance of these drug crystal/HA hydrogels for IA therapy. HA molecules are fairly stable in hyaluronidase‐free solutions, but are quickly catabolized in synovial fluid, which is primarily attributed to the existence of reactive oxygen species in synovial fluid.^39^ In vivo degradation of HA in synovial fluid is a complex process, involving the diffusion of transition metals and their carrier proteins, and the generation of unstable reactive oxygen species,^39^ which is challenging to be replicated in vitro or ex vivo. In a pilot study, after mixing HA with synovial fluid at 37°C for overnight, no detectable decrease in HA molecular weight was observed (Figure S5). We then evaluated the HA hydrogel stability in vivo in healthy Sprague Dawley (SD) rat joints. A 50 μl of hydrogel was intra‐articularly injected. The fluorescence intensity of Alexa Fluor 647‐labeled HA within the region‐of‐interest (ROI) was measured over 28 days using an In Vivo Imaging System (IVIS), as shown in Figure 4b. Total radiant efficiency (RE) values within the ROI were used to evaluate the remaining hydrogels over time. The hydrogel resided inside the joint for more than 4 weeks, with a gradual decrease in RE values over time. After 28 days, ~35% of the injected fluorophore remained in the synovial fluid, with an averaged RE value ~7 times higher than that from the control group, shown as the background line in Figure 4c. Compared to uncross‐linked HA polymers with typical joint residence time of ~12 to 24 h,^14^ this HA hydrogel resided within the joint space for a much longer period. The long joint half‐life of the HA hydrogel is of potential clinical interest for other IA therapies, although the long‐term safety profile of the hydrogel and its degradation products in healthy joints requires further evaluation.

### 2.4 Design of drug nanocrystals formulations and in vitro release study

**FIGURE 5:**
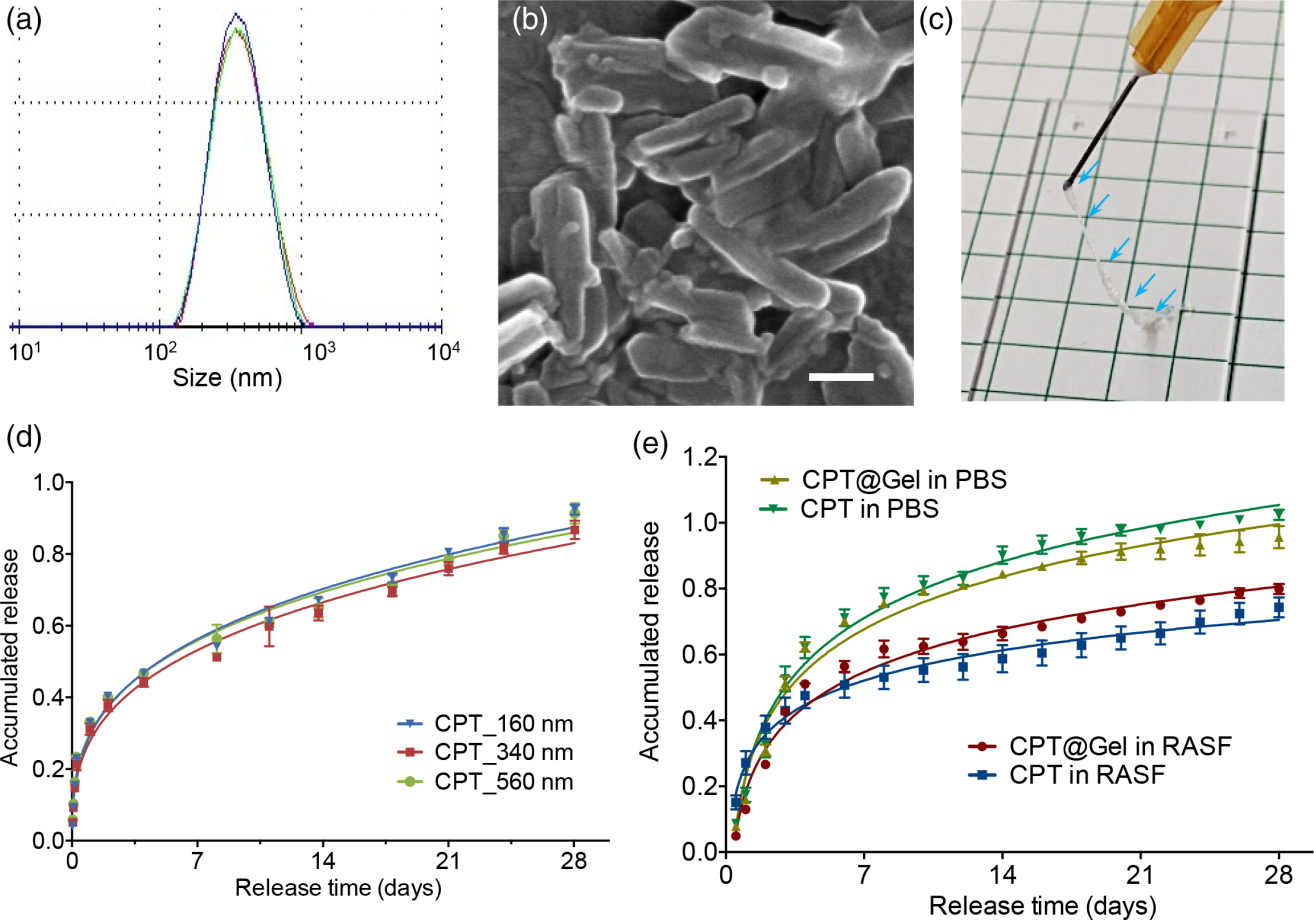
CPT nanocrystals formulations for in vitro drug release study. Typical size distribution (a, *n* = 3) and morphology (b) of CPT nanocrystals (scale bar = 250 nm). (c) Image of CPT nanocrystal encapsulated hydrogels after injection using 26‐gauge needles. In vitro dissolution of CPT nanocrystals with three different sizes (160, 340, and 560 nm) from their corresponding gel formulations in PBS (d) and RA synovial fluids (RASF) (e) at 37°C. Each value represents the mean ± SD (*n* = 3)

Crystalline CPT was formulated to demonstrate the sustained release behavior from such drug nanocrystal/HA hydrogel depots. The size of drug crystals was controlled within the submicron range by manipulating the process parameters such as drug concentration, solvent/antisolvent ratio, temperature, and precipitation time. The size and morphology of drug nanocrystals were characterized by dynamic light scattering (DLS) and scanning electron microscopy (SEM). Figure 5a,b and Figure S6 show the typical size distribution and morphology of the CPT drug crystals. These crystals were suspended in water, mixed with HA‐CBT solution, and cross‐linked by the addition of Cys‐PEG_46_‐Cys solutions. Their injectability after encapsulation was verified (Figure 5c).

We firstly evaluated the dissolution rate of CPT nanocrystals by incubating the CPT nanocrystals in phosphate‐buffered saline (PBS) at 37°C. A slow and sustained dissolution behavior was observed with around 80% of CPT dissolved over 28 days. A similar sustained drug release was observed with the CPT nanocrystal‐encapsulated hydrogels (Figure 5d,e). We also found that the drug nanocrystals with size in the range between 160 and 560 nm (Figure S6) have a similar dissolution rate either in naked or encapsulated forms, which gives flexibility for the nanocrystal preparation.

To validate the slow release for IA therapy, dissolution was also examined in RA synovial fluid (RASF) to simulate the inflamed joint environment. Compared with the release in PBS, CPT has a slower release rate with around 70% released within 28 days (Figure 5e). This could be attributed to the high viscosity of RASF in the release medium which hindered the free drug diffusion out of the dialysis membrane. The slow drug release rate further highlights the potential of such hydrogel/drug nanocrystals formulation for IA‐localized drug delivery.

### 2.5 Therapeutic efficacy in an arthritic rat model

**FIGURE 6:**
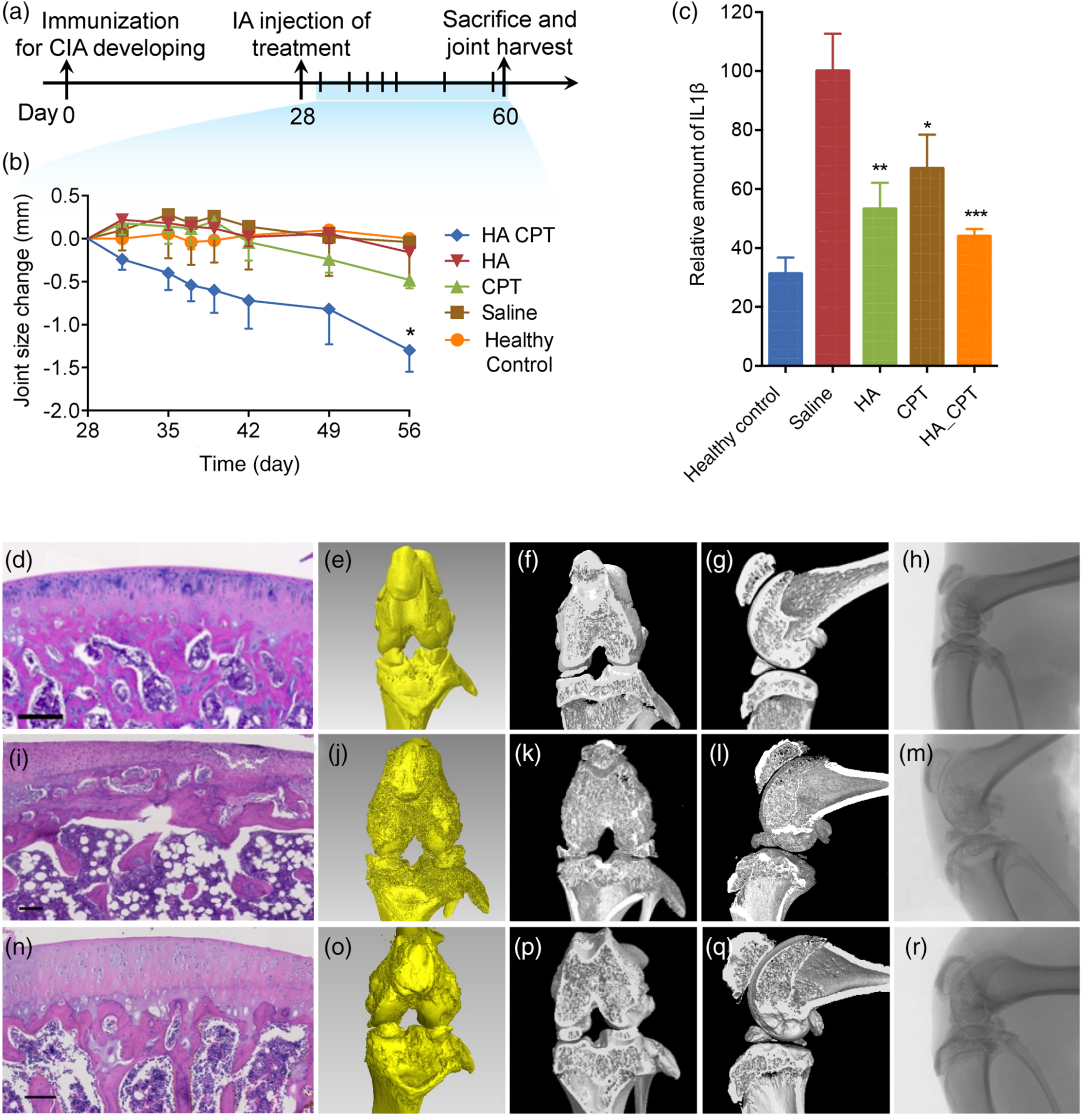
Therapeutic efficacy in collagen‐induced arthritis animal models. (a) Experimental outline: arthritis was induced by intradermal injection of complete Freund's adjuvant/collagen II emulsion on day 0. On day 28, CPT nanocrystal encapsulated HA hydrogels (HA‐CPT), blank HA hydrogels (HA), free CPT nanocrystals (CPT), and saline was injected and joint size was monitored for another 4 weeks. Animals were sacrificed on day 60 with joints being harvested. (b) Change in joint size was measured by clippers. All data are presented as mean ± *SEM* (*n* = 5) and statistical analysis by *t* test (*p < 0.05). (c) The relative amount of IL‐1β in joint homogenates. All data are presented as mean ± *SEM* (*n* ≥ 4) and statistical analysis by one‐way ANOVA (*p < 0.05, **p < 0.01, ***p < 0.001). Histological analysis with H&E staining (d, i, n) and microcomputed tomography evaluation with representative reconstructed three‐dimensional images (e, j, o), coronal images (f, k, p), sagittal images (g, l, q), and X‐ray projection images (h, m, r) of dissected joint tissues of healthy control (d–h), saline‐treated group (i–m), and HA‐CPT‐treated group (n–r) (scale bar = 100 µm)

Having shown the long‐term stability of HA hydrogels and a sustained release of the encapsulated drug nanocrystals, we then validated the potential of CPT nanocrystal‐loaded HA hydrogel for IA therapy. To this end, a collagen‐induced arthritis (CIA) model was developed by intradermal injection of complete Freund's adjuvant/collagen II emulsion to SD rats.^40^, ^41^, ^42^ The incidence of arthritis was confirmed by the erythema and swelling of the paw joints (Figure S7). Three types of formulations were prepared, including CPT nanocrystal‐encapsulated HA hydrogels (HA‐CPT), blank HA hydrogels (HA), and free CPT nanocrystals (CPT) with saline as the placebo. On Day 28 of arthritis induction, each formulation was injected intra‐articularly (Figure 6a). Treatment with HA‐CPT reduced arthritis severity compared to those treated by blank HA hydrogels or saline (Figure 6b). Free CPT nanocrystals did not show a significant reduction of joint size compared to saline‐treated animals, which could be attributed to the rapid joint clearance of the sub‐micron‐sized agents.^15^ The local therapeutic effect on inflammatory arthritic joints was further evaluated by quantifying the inflammatory cytokine interleukin‐1β (IL‐1β) and interleukin‐6 (IL‐6) in the joint homogenate. The IL‐1β level showed a maximum value for the saline‐treated group, followed by the free CPT nanocrystals, blank HA hydrogels, and the HA‐CPT formulation (Figure 6c). Similarly, the IL6 level in HA‐CPT formulation is significantly lowered comparing to the saline‐treated group (Figure S8). Histological and microcomputed tomography (micro‐CT) analysis of the dissected joint tissues at Day 60 further confirmed the therapeutic effect of HA‐CPT formulation. Compared with the healthy control (Figure 6d–h; Figure S9k), knee joints of CIA animals (saline group) harvested at Day 60 showed severe joint destruction with significant erosion of cartilage and bone as shown in Figure 6i–m and Figure S9l. However, in the HA‐CPT‐treated group, the bone and cartilage damage were alleviated accompanied by a smooth cartilage surface (Figure 6n–r; Figure S9m). No obvious reduction in the cartilage damages were observed in the HA‐ and CPT‐treated control groups (Figure S9a–j,n,o).

An attempt to extract and quantify the plasma CPT concentrations at different time points from Day 1 to Day 32 after treatment was made by using an ultrahigh‐performance liquid chromatography (HPLC)/mass spectrometer with 7‐ethyl‐10‐hydroxyCPT as internal standard (LOD of 6 ng/ml and recovery of ~76.87 ± 2.10%), but no free CPT was detected. This could be attributed to the slow dissolution rate of the CPT nanocrystals and the rapid plasma clearance rate, giving an extremely low plasma CPT concentration. This indeed is highly desirable for localized IA therapy as such low plasma CPT concentration minimizes the systemic toxicity. Histology of the heart, lungs, liver, kidney, and spleen are normal in all groups as shown in Figure S10. No body‐weight reduction was observed for any of the treated animals (Figure S11).

## 3 DISCUSSION

There is an unmet medical need for developing new treatments for inflammatory arthritis (e.g. RA). Therapeutics that can inhibit the tumor‐like aggressive proliferation of RA synoviocytes are potentially effective in treating RA.^9^ However, the use of antiproliferative drugs, commonly used for cancer treatments, to treat RA is greatly limited by their systemic toxicity and low joint bioavailability.^14^ CPT, as chosen here, is one such antiproliferative agent with antiarthritic potentials.^12^, ^13^ To overcome the formulation and delivery challenges, we formulated CPT into nanocrystals, encapsulated them into an injectable HA hydrogel, and intra‐articularly injected it into the inflamed joints to achieve localized and sustained drug delivery.

We developed an in situ click‐cross‐linkable, injectable HA hydrogel using a biocompatible click reaction: CBT/cysteine reaction, which is the last step in the generation of D‐luciferin in fireflies.^30^ We optimized the gel modulus, a key parameter related to its internal structure and thus important for regulating drug crystal release, by tuning the degree of modification of CBT, cysteine linker size and polymer concentration. Remarkably, the HA hydrogel can reside inside the rat joint for more than 4 weeks with ~35% of hydrogels remaining on day 28, which is much longer comparing to the uncross‐linked HA polymer (~12 to 24 h^14^).

The HA hydrogel described here can encapsulate CPT nanocrystals in a simple admix manner and is readily injectable to form a drug depot locally. In vitro release study confirmed that CPT can slowly release in either PBS or RASF for 4 weeks. The promise of this CPT nanocrystal/HA hydrogel formulation in achieving long‐term‐localized IA drug delivery was further highlighted in vivo using a CIA model in rats. Animals treated with the experimental formulation showed a reduction in arthritis severity.

It is important to note the limitations of this study. In this work, we did not detect any circulating CPT molecules in the plasma at different time points, thus future animal studies should incorporate experiments to evaluate the plasma and joint pharmacokinetic profiles using radiolabeled drugs. Systemic biodistribution of these radiolabeled drugs could also be studied. Also, rats were sacrificed for efficacy and toxicity evaluation at Day 60 and found no signs of organ toxicity. It would be necessary to evaluate the histotoxicity and cartilage destruction at different time points.

In summary, we have developed an effective drug nanocrystal‐encapsulated HA hydrogel system for IA therapy. This drug nanocrystal/HA hydrogel system can potentially advance the field of antirheumatic drug development and accelerate the discovery of new treatments to treat RA.

## 4 EXPERIMENTAL SECTION

### 4.1 Materials

HA (250 kDa) was purchased from Creative PEGWorks and used as received. Immunization Grade Chick Type II Collagen and Complete Freund's Adjuvant were obtained from Chondrex INC. IL‐1β Rat ELISA Kit, Alexa Fluor 647 hydrazide was obtained from Life Technologies. Human RASF was obtained from BioIVT. All other chemicals are obtained from Sigma Aldrich and used without further purification.

### 4.2 Synthesis of HA‐CBT conjugates

HA (250 kDa) was first dissolved in 1:1 DMSO/Milli‐Q water with a concentration of 10 mg/ml. Sulfo‐NHS (1 equiv. to Gly‐CBT) and EDC•HCl (1 equiv. to Gly‐CBT) were dissolved in Milli‐Q water and 1:1 DMSO/Milli‐Q water, respectively, and added to the HA solution immediately after preparation. The reaction mixture allowed to stir for 2 h at room temperature. For the targeted DC of 10%, 18 mmol% of Gly‐CBT (relatively to disaccharide units) was dissolved in DMSO and added to the reaction. After overnight reaction at room temperature, the conjugate was purified via precipitation into ethanol/PBS solution, and dialysis against Milli‐Q water (3 times water change), followed by lyophilization for 3 days. A similar procedure was followed for synthesizing HA‐CBT conjugates with different DC by changing the feed amount of Gly‐CBT. To synthesize Alexa Flour 647‐labled HA, 100 mg HA was dissolved in 10 ml Milli‐Q water, mixed with 1.5 mg EDC•HCl and 1 mg Alexa Flour 647 aqueous solution at room temperature for overnight, and purified and lyophilized in a similar manner. Gly‐CBT was then conjugated to Alexa Flour 647‐labeled HA in the same procedure to achieve the Alexa Flour 647‐labeled HA‐CBT conjugates.

### 4.3 Synthesis of Cys‐PEG_n_‐Cys linkers

Cys‐PEG_*n*_‐Cys linkers with *n* = 0, 8, and 46 were all synthesized using a similar procedure. For Cys‐PEG_46_‐Cys synthesis, N‐(tert‐Butoxycarbonyl)‐S‐trityl‐L‐cysteine (Boc‐Cys[Trt], 500 mg, 1.08 mmol) was activated by CDI (218.9 mg, 1.35 mmol) in anhydrous DMF (5.91 ml) for 30 min under nitrogen. Poly(ethylene glycol) diamine (average *M*_n_ = 2000, 900 mg, 0.45 mmol) in anhydrous DMF (5.4 ml) was added to the reaction mixture, reacted for another 4 h at room temperature. After removing the DMF via rotary evaporation, the product was dissolved in DCM, washed by Milli‐Q water and brine, dried by MgSO_4_, and concentrated via rotary evaporation. The crude product was further purified through repeated precipitation and washing steps in cold hexane/diethyl ether (1:1) mixtures. Deprotection was achieved with a solution of TFA/water (97/3) in the presence of triisopropylsilane as a scavenger. The product was then isolated through prep‐HPLC (total yield 43%). The same molar feed ratio was used for the synthesis of other linkers.

### 4.4 CPT nanocrystal preparation

CPT nanocrystals were prepared using an antisolvent precipitation method under sonication, as reported previously.^43^ Crystal size was tuned by modifying the preparation conditions. Briefly, 5 mg of CPT was dissolved in 5 ml dimethyl sulfoxide (DMSO) and filtered by a 0.22‐μm filter. This solution was slowly added into 50 ml of 0.1% α‐tocopherol aqueous solution (pH 4.0). Crystallization proceeded under stirring (500–700 rpm) and sonication in an ice/water bath for an hour, yielding crystals with size of ~340 nm (Figure S6). To prepare crystals with smaller size (~160 nm; Figure S6), CPT/DMSO solution with CPT concentration of 5 mg/ml and 1% α‐tocopherol aqueous solution (pH 4.0) were used. Stirring and sonication at room temperature for 2 h were carried out to prepare crystals with larger size (~560 nm; Figure S6). The obtained CPT nanocrystals were collected by centrifugation and washed with Milli‐Q water (pH 4.0).

### 4.5 Hydrogel fabrication with or without drug nanocrystals

All hydrogels were prepared in a similar manner. For a typical procedure of blank hydrogels without drug nanocrystals, HA‐CBT with DC of 10% was dissolved in saline at a concentration of 20 mg/ml and mixed thoroughly with freshly prepared Cys‐PEG_46_‐Cys stock solution (57 mg/ml) at an equivalent molar ratio of CBT to Cysteine. For the CPT nanocrystal‐loaded hydrogel preparation, CPT nanocrystal suspension in saline (with CPT concentration of 7.43 mg/ml) were firstly mixed with HA‐CBT solution (20 mg/ml). Freshly prepared Cys‐PEG‐Cys solution (57 mg/ml) was added and mixed immediately.

### 4.6 Proton nuclear magnetic resonance ( ^1^H NMR)

The HA‐CBT conjugates and Cys‐PEG‐Cys linkers were characterized by ^1^H NMR using an Agilent DD2 600‐MHz NMR Spectrometer (Santa Clara, CA) with MestReNova (version 10.0.1) processing software. The HA‐CBT conjugates were dissolved in deuterated water (D_2_O). The chemical shifts were represented in parts per million (ppm), with reference to the signal for D_2_O at 4.79 ppm. The degree of Gly‐CBT conjugation (DC) was calculated by ^1^H NMR of the conjugates as Equation (1).

(1) $$\mathrm{DC}=\frac{I\left(a+b+c\right)/3}{I\left(d\right)/3}\times 100\%$$

Similarly, Cys‐PEG‐Cys linkers and their protected forms were dissolved in D_2_O and acetone‐*d*_6_, respectively, and their chemical shifts were reported with reference to the solvent peak.

### 4.7 Gel permeation chromatography

HA‐CBT conjugates were analyzed by a Viscotek 270max GPC system (Malvern Instruments Ltd., UK) equipped with an autosampler, a TDA 305 triple detection system, a UV detector 2600, a pump, a degasser, and OmniSec software. A combination of two TSKgel® GPC columns (G4000PW_XL_ and G3000PW_XL_, 30 cm L × 7.8 mm ID) was eluted with triple‐filtered 0.2 M NaNO_3_ and 0.01 M NaH_2_PO_4_ (pH 6.8) aqueous solution at a flow rate of 0.6 ml/min at 35°C. Samples were dissolved in the mobile phase solution and filtered through a 0.22 μm Acrodisc® syringe filter (Waters Corp., Milford, MA). A 100 μl of the polymer solutions was injected for each run. UV absorbance at 330 nm was used to confirm the conjugation of Gly‐CBT to HA.

### 4.8 MALDI‐TOF‐MS

Cys‐PEG‐Cys linkers were characterized by MALDI‐TOF‐MS using a Bruker UltrafleXtreme MALDI‐TOF mass spectrometer. 2,5‐Dihydroxybenzoic acid matrix was dissolved in acetonitrile/Milli‐Q/trifluoroacetic acid (volume ratio 50/50/0.1) at a concentration of 20 mg/ml. A 1 μl of sample solution (8 mg/ml) was mixed with 1 μl of the matrix solution and deposited onto a MALDI sample plate. Samples were allowed to dry in air and run in linear mode.

### 4.9 Rheological characterization

The rheological properties of hydrogels were measured by an AR‐G2 Rheometer (TA instruments, Newcastle, DE), equipped with a Peltier heating system and an environmental enclosure for temperature control. For the gelation kinetic test, the premixed solution was quickly transferred to the bottom plate and the storage (*G*′) and loss (*G*″) moduli were monitored using a 20 mm parallel plate at 37°C under 1% strain and 1.59 Hz. Strain sweep was performed from 0.01% to 10% at 1.59 Hz to confirm the linear viscoelastic regime. Frequency sweep experiments were performed from 0.01 to 10 rad/s at 1% strain. Gel samples were prepared either on a customized mold or the plate in situ. A conventional solvent trap was used to minimize the evaporation of the solvent. Care was taken to ensure reproducibility and all results reported were averaged over three runs.

### 4.10 Dynamic light scattering

Crystal size was analyzed using a Zetasizer Nano Zs (Malvern Instruments Ltd., UK) with a 633‐nm wavelength laser. Malvern Zetasizer software was used for DLS system control, data acquisition, and processing. All results reported were averaged over three runs.

### 4.11 Scanning electron microscopy

Crystal morphology was studied with SEM. Diluted crystal solution was deposited on an aluminum stub (Ted Pella, Inc., Redding, CA) and allowed to dry in air. After coating with ~10 nm of Platinum (Pt)/Palladium (Pd) (80/20) alloy using 150T S metal sputter coater (Quorum Technologies Ltd., UK), samples are imaged using the Ultra Plus Field Emission Scanning Electron Microscope (Zeiss, Germany) with 5 KV acceleration voltage.

### 4.12 High‐performance liquid chromatography

All the HPLC analysis was performed on an Agilent 1200 series HPLC instrument (Agilent Technologies, USA) equipped with an autosampler, a LC binary pump, a thermostatic column compartment, a diode array detector (DAD) and the Agilent ChemStation software. An Agilent C18 column was used for the chromatographic separation. The flow rate was set at 0.5 ml/min, Elution conditions were set as: mobile phase A: 0.1% TFA in acetonitrile and B 0.1% TFA in Milli‐Q water; gradient over 10 min from A/B = 95:5 to 0:100. For standard curves, CPT stock solution (125 μg/ml in DMSO) was diluted by a factor of 2 for 10 times. Twenty microliters of samples were injected for each run and detected by DAD at 370 nm.

### 4.13 Fluorescence recovery after photobleaching

To investigate the mobility of encapsulated nanoparticles, the hydrogels were loaded with FITC‐dextran (70 kDa) or commercial polystyrene nanoparticles with a size of 50 and 100 nm (Fluoresbrite® Yellow Green Microspheres, Polysciences, Inc., USA). FRAP experiments were performed on a confocal laser scanning microscope (Zeiss LSM 710, Zeiss, Germany) by setting the 488‐nm argon laser to 90% power and using the 10× objective lens with completely opened pinhole. Twenty microliters of hydrogels were placed on a glass slide and covered with a coverslip. A region with an area of 886 μm^2^ was bleached by the maximum laser intensity. Pre‐ and post‐bleach images were taken by with a laser intensity of 0.2% transmission. The data analysis method was adapted from previous work.^44^

### 4.14 In vitro release study

A 50‐μl CPT‐loaded HA hydrogels was soaked in 1 ml PBS at 37°C under gentle rotating. A 0.5 ml of release medium was taken routinely (up to 30 days) and refilled with 0.5 ml of fresh PBS. Aliquots of 200 μl were used for HPLC analysis. The in vitro release in RASF was performed by using a slight modification of a previously described procedure.^45^ Briefly, 50‐μl CPT‐loaded HA hydrogels were placed in dialysis tubing (10 kDa molecular weight cutoff) and suspended in 650 μl PBS. A 200 μl human RASF or an equal amount of PBS was added to the dialysis bag. The dialysis bags were placed in 20 ml PBS and incubated at 37°C under gentle shaking. One milliliter of the medium was taken out routinely (up to 30 days) and analyzed using HPLC as described above.

### 4.15 Preparation and treatment of CIA animal model

All in vivo experiments were performed in accordance with protocols approved by the Institutional Animal Care and Use Committee (IACUC) of Harvard University. Rats were housed in a standard 12 h light/dark cycle conditions and fed with chow and water ad libitum. CIA model was developed according to a previous protocol.^40^, ^46^ Chick type II collagen (2 mg/ml in 0.01 M acetic acid) was emulsified with an equal volume of complete Freund's adjuvant using a homogenizer (T10 basic, IKA Works, Inc., GE). A 200 μl of the emulsion was intradermally injected into the base of the tail on experimental Day 0. The incidence of arthritis was monitored two to three times per week for 4 weeks by measuring the swelling of joints and digits. On Day 28, five arthritic animals at a mean body weight of 325 g were randomized into each of four treatment groups: saline, CPT crystals, HA hydrogels, and CPT crystal‐loaded HA hydrogels (HA‐CPT). Fifty microliters of control or experimental formulations (containing 1.3 mg CPT/ml and/or 15 mg HA/ ml) were injected at one joint per animal using a 26 G × 1/2″ disposable needle. Arthritis severity and body weight were measured 2 to 3 days per week for another 4 weeks and sacrificed at Day 60.

### 4.16 IVIS imaging

Alexa Fluor 647‐labeled HA was used for the fabrication of HA hydrogels. After IA injection, an IVIS (Spectrum, PerkinElmer, USA) with an anesthesia system and Living Image software (3.1, Caliper, USA) was used to image and quantify the fluorescence of joint over 28 days.

### 4.17 Joint homogenization and protein retrieval

After euthanizing, treated joints with surrounding synovial tissues were dissected and frozen in liquid nitrogen, pulverized by a mortar and pestle, and homogenized in a lysis buffer (89901, Thermo Scientific, USA) containing protease and phosphatase inhibitors (78442, Thermo Scientific, USA) and centrifuged for 10 min at 400 g (4°C) to collect the supernatants. IL‐1β concentration was quantified by ELISA (BMS630, Thermo Scientific, USA).

### 4.18 Microcomputed tomography

Animal knee joints were analyzed by micro‐CT using the Nikon Metrology HMX ST 225 CT scanner (Nikon Metrology, UK) with voxel size of 16.08–49.26 μm (500 ms exposure time, 65–85 kV energy source, 115–145 mA current). Approximately, 3000 projections were acquired over a rotation range of 360°, with a 0.12° rotation step. 3D reconstruction of representative regions of articular knee joints was created by VG studio MAX 3.0.1 software (Volume Graphics, Germany).

### 4.19 Histological analysis

Retrieved samples were fixed in 10% neutral buffered formalin, embedded in paraffin, and sectioned according to standard histological process. Slides were then stained with hematoxylin and eosin (H&E) and safranin‐O.

### 4.20 Statistics analysis

All data were reported as means ±*SEM* from separate experiments. Statistical analyses were performed by *t*‐test or one‐way ANOVA methods using GraphPad Prism (GraphPad Software, USA). Differences were considered statistically significant when *p* < 0.05.

## AUTHOR CONTRIBUTIONS

**Yongsheng Gao:** Conceptualization; formal analysis; methodology; validation; writing ‐ original draft; writing‐review & editing. **Douglas Vogus:** Conceptualization; methodology. **Zongmin Zhao:** Data curation; investigation. **Wei He:** Methodology. **Vinu Krishnan:** Methodology. **Jayoung Kim:** Methodology; visualization. **Yujie Shi:** Methodology. **Apoorva Sarode:** Methodology. **Anvay Ukidve:** Methodology. **Samir Mitragotri:** Conceptualization; resources; supervision.

## Supporting information


**Appendix** S1: Supporting information
