# Supplementary material for: Injectable hyaluronic acid hydrogels encapsulating drug nanocrystals for long‐term treatment of inflammatory arthritis
Source: Bioeng Transl Med. 2021 Sep 15;7(1):e10245. doi: 10.1002/btm2.10245 (PMC8780912; doi:10.1002/btm2.10245)
Supplement: Supplementary file 1 — Appendix S1: Supporting information [file BTM2-7-e10245-s001.docx]

Supporting Information

Hyaluronic acid hydrogels with drug nanocrystals for long-term treatment of inflammatory arthritis

Yongsheng Gao, Douglas Vogus, Zongmin Zhao, Wei He, Vinu Krishnan, Jayoung Kim, Yujie Shi, Apoorva Sarode, Anvay Ukidve, Samir Mitragotri*

**Method**

Synthesis of Gly-CBT

*N*-(tert-Butoxycarbonyl)glycine (Boc-glycine, 432 mg, 2.47 mmol) was first reacted with TBTU (786 mg, 2.47 mmol) in anhydrous DMF (9.6 mL) and DIPEA (0.64 mL) for 30 mins at room temperature. 6-amino-2-cyanobenzothiazole (300 mg, 1.71 mmol) was dissolved in anhydrous DMF (3 mL) with DIPEA (0.64 mL), and added to the above reaction solution. The reaction mixture was allowed to stir for another 4 hrs. After removing the DMF via rotary evaporation, the product was redissolved in ethyl acetate, washed with Milli-Q water (x1), saturated NaHCO_3_ (x2) and brine (x1), dried over MgSO_4_ and concentrated. The crude product was purified using silica column chromatography (3:1 hexane/ethyl acetate). The as-prepared compound was deprotected in a 1:1 TFA/DCM solution for 1.5 hrs, before subjected to prep-HPLC purification. (Total yield 45-55%). LC-MS calcd m/z for [M+H]^+^: 233.04, found: 233.1. ^1^H NMR (500 MHz, Acetone-*d*_6_) δ 8.76 (d, *J* = 2.0 Hz, 1H), 8.17 (d, *J* = 9.0 Hz, 1H), 7.81 (dd, *J*_1_ = 2.1 Hz, *J*_2_ = 9.0 Hz, 1H), 4.90 (s, 2H).

Figure S1. UV absorbance of HA-CBT. a. GPC UV 3D spectra. b. UV absorbance spectrum.

Figure S2. ^1^H NMR (acetone-*d*_6_, 600 MHz) spectrum of N-(tert-Butoxycarbonyl)-S-trityl-L-cysteine modified PEG linkers with different molecular weights (*before deprotection*).

Figure S3. ^1^H NMR (D_2_O, 600 MHz) spectrum of Cys-PEG_n_-Cys linkers with different molecular weights (*after deprotection*).

Figure S4. Swelling test of HA hydrogel in PBS at 37 ºC. Swelling ratio was calculated by the mass increasement of hydrogel to its original weight: (*W_t_*-*W*_0_)/*W*_0_.

Figure S5. GPC RI profiles of native HA after incubation in PBS, RASF and H_2_O_2_ overnight.


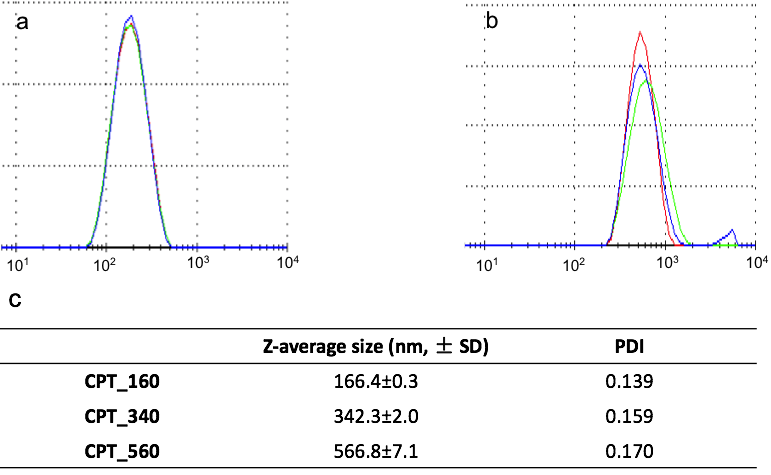


Figure S6. Intensity particle size distribution of CPT nanocrystals. a. CPT_160, b. CPT_560, c. averaged particle size of CPT nanocrystals. The data were collected 3 x 10 runs for each sample.

Figure S7. The erythema and swelling of the paw of rat at Day 28, compared with that at Day 0.

Figure S8. The relative amount of IL-6 in joint homogenates. All data are presented as mean ± SEM and statistical analysis by one-way ANOVA(**p* < 0.05)

Figure S9. Histological analysis with H&E staining (a, f) and micro-computed tomography evaluation with representative reconstructed three-dimensional images (b, g), coronal images (c, h), sagittal images (d, i) and X-ray projection images (e, j) of dissected joint tissues of CPT-treated group (a~e) and HA treated group (f~j). Safranin-O staining of the knee joints of healthy rats (k), and arthritic rats treated with saline (l), HA-CPT (m), CPT(n) and HA (p). (scale bar: 100 nm)


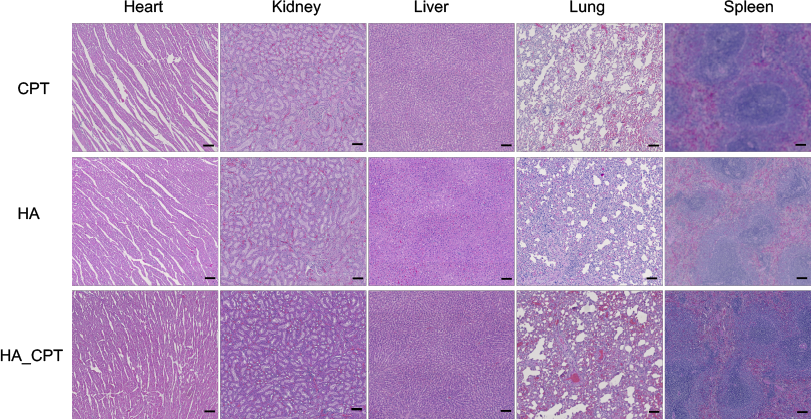


Figure S10. Representative H&E sections of major organs (heart, kidney, liver, lung, and spleen) from rats treated with CPT, HA, and HA-CPT. (scale bar: 100 nm)

Figure S11. Body weight changes of rats from Day 0 ~ 56.

Calculation of mesh size [1]:

By using the blob model, the mesh size ξ is related to the blob size $\xi_{b}$ as

$\xi\approx{Q^{1/3}\xi_{b}\approx Q^{1/3}R}_{g}{(\frac{c}{c^{*}})}^{-\nu/(3\nu-1)}$ (1)

Where *R*_g_ is the radius of gyration, *c* is the polymer concentration, *c** is the overlap concentration, and *ν* is the Flory’s exponent. *Q* is the swelling ratio from as-prepared state to swollen state.

By using Brannon-Peppas model, the mesh size ξ is related to the molecular weight between cross-links *M*_c_ as

$\xi\approx Q^{1/3}k{M_{c}}^{\nu}$ (2)

Where *k* is a constant, *M*_c_ is calculated based on storage modulus *G*’ as

$\frac{1}{M_{c}}=\frac{G'}{RTc}+\frac{2}{\bar{M_{n}}}$ (3)

Where *c* is the polymer concentration, R is the gas constant, T is the temperature and *M*_n_ is the molecular weight of HA.

Table S1. Values are used for the calculation for calculation the

|  | Value | Unit | Source |
| --- | --- | --- | --- |
| R_g_ | 47 | nm | Measured via GPC Triple Detection |
| c | 20 | mg/mL | Experimental value |
| c* | 9.5 | mg/mL | Measured via GPC Triple Detection |
| v | 0.6 |  | [2] |
| G’ | 0.221 | kPa | Rheology |
| R | 8314.463 | L⋅Pa⋅K^−1^⋅mol^−1^ |  |
| T | 298 | K |  |
| M_n_ | 181712 | g/mol | Measured via GPC Triple Detection |
| k | 0.028 |  | [2] |

Reference

[1] Y. Yu, Y. Chau. *Biomacromolecules* **2015**, 16: 56-65.

[2] R. Mendichi, L. Šoltés, A. G. Schieroni. *Biomacromolecules.* 2003, 4: 1805-1810.
